# Laboratory policies and practices for thyroid function tests in Croatia: survey on behalf of Working Group for Laboratory Endocrinology of the Croatian Society of Medical Biochemistry and Laboratory Medicine

**DOI:** 10.11613/BM.2022.030702

**Published:** 2022-08-05

**Authors:** Adriana Bokulić, Ivana Zec, Sanja Goreta, Nora Nikolac Gabaj, Marija Kocijančić, Tihana Serdar Hiršl, Anamarija Đuras, Mateja Troha, Lada Stanišić, Daniela Šupe-Domić, Sanda Jelisavac Ćosić, Koraljka Đurić, Domagoj Marijančević, Marija Siter Kuprešanin, Iva Lukić, Alenka Pezo, Jasna Leniček Krleža

**Affiliations:** 1 Laboratory of Endocrinology, Department of Oncology and Nuclear Medicine, Sestre Milosrdnice University Hospital Center, Zagreb, Croatia; 2 University Department of Chemistry, Sestre Milosrdnice University Hospital Center, Zagreb, Croatia; 3 Faculty of Pharmacy and Biochemistry, University of Zagreb, Zagreb, Croatia; 4 Central Laboratory, University Hospital Halle, Halle (Saale), Germany; 5 Medical Biochemistry Laboratory, Synlab Hrvatska-Polyclinic for Medical Laboratory Diagnostics, Zagreb, Croatia; 6 Department of Medical Biochemistry Laboratory, General Hospital Varaždin, Varaždin, Croatia; 7 Department of Laboratory Diagnostics, General Hospital Dr. Josip Benčević, Slavonski Brod, Croatia; 8 Department of Medical Laboratory Diagnostics, University Hospital of Split, Split, Croatia; 9 Department of Health Studies, University of Split, Split, Croatia; 10 Department of Nuclear Medicine and Radiation Protection, University Hospital Centre Zagreb, Zagreb, Croatia; 11 Medical Biochemistry Laboratory, Special Hospital AGRAM, Zagreb, Croatia; 12 Department of Medical Laboratory Diagnostics, University Hospital Sveti Duh, Zagreb, Croatia; 13 Department for Clinical Laboratory Diagnostics, University Hospital Centre Osijek, Osijek, Croatia; 14 Medical Biochemistry Laboratory, Primary Health Care Centre Zagreb - East, Zagreb, Croatia; 15 Croatian Society of Medical Biochemistry and Laboratory Medicine (CROQALM), Zagreb, Croatia; 16 Department of Laboratory Diagnostics, Children’s Hospital Zagreb, Zagreb, Croatia

**Keywords:** thyroid function test, standardization, survey

## Abstract

**Introduction:**

Laboratory plays important part in screening, diagnosis, and management of thyroid disorders. The aim of this study was to estimate current laboratory preanalytical, analytical and postanalytical practices and policies in Croatia.

**Materials and methods:**

Working Group for Laboratory Endocrinology of the Croatian Society of Medical Biochemistry and Laboratory Medicine designed a questionnaire with 27 questions and statements regarding practices and protocols in measuring thyroid function tests. The survey was sent to 111 medical biochemistry laboratories participating in external quality assurance scheme for thyroid hormones organized by Croatian Centre for Quality Assessment in Laboratory Medicine. Data is presented as absolute numbers and proportions.

**Results:**

Fifty-three participants returned the questionnaire. Response rate varied depending on question, yielding a total survey response rate of 46-48%. All respondents perform thyroid stimulating hormone (TSH). From all other thyroid tests, most performed is free thyroxine (37/53) and least TSH-stimulating immunoglobulin (1/53). Laboratories are using nine different immunoassay methods. One tenth of laboratories is verifying manufacturer’s declared limit of quantification for TSH and one third is verifying implemented reference intervals for all performed tests. Most of laboratories (91%) adopt the manufacturer’s reference interval for adult population. Reference intervals for TSH are reported with different percentiles (90, 95 or 99 percentiles).

**Conclusion:**

This survey showed current practices and policies in Croatian laboratories regarding thyroid testing. The results identified some critical spots and will serve as a foundation in creating national guidelines in order to harmonize laboratory procedures in thyroid testing in Croatia.

## Introduction

Thyroid disorders are relatively common and widespread health problem. Among Europeans, 11% have some type of thyroid dysfunction, yet only half of them are diagnosed. Misdiagnosis and mismanagement of thyroid disorders do happen, and they should not be ignored. Quality improvements of all the procedures and protocols must be imperative (*1*).

Together with medical history, physical exams and thyroid imaging, laboratory tests are integral part of screening, diagnosis, and management of thyroid disorders. Laboratory assays for thyroid dysfunction include general thyroid function tests as thyroid stimulating hormone (TSH), free thyroxine (fT4), total thyroxine (T4), free triiodothyronine (fT3) and total triiodothyronine (T3) and more specialized tests as thyroid peroxidase antibody (anti-TPO), thyroglobulin antibody (anti-Tg), thyroglobulin (Tg), calcitonin (hCT), TSH receptor antibody (TRAb) and TSH-stimulating immunoglobulin (TSI) (*2*). According to Croatian Centre for Quality Assessment in Laboratory Medicine (CROQALM), TSH is performed in laboratories on all levels of health care settings in more than 100 laboratories across country (*3*). Accurate, reliable, and comparable measurement in laboratory medicine is achieved through standardization and/or harmonization. Common thyroid tests, as TSH, are available on the market for several decades. Still, laboratories are facing many challenges as incomparable results among different immunoassay platforms or method interferences. Consequently, standardization and/or harmonization of thyroid function tests still remain obstacle that is difficult to overcome (*4*).

Current national and international guidelines in the field of thyroid disorders refer more on clinical aspects of disorders and less on laboratory (*5*–*7*). At this moment, there are initiatives to standardize and harmonize most commonly used thyroid functional tests by International Federation of Clinical Chemistry, but none of them entered into routine laboratory work (*8*–*10*).

Croatian Society of Medical Biochemistry and Laboratory Medicine (CSMBLM) established a Working Group (WG) for Laboratory Endocrinology. The first task of the WG is to explore current practices regarding thyroid disorder assays in Croatian laboratories and to identify major problems. The main goal of the WG is to make recommendations tailored to Croatian laboratories taking into account their current protocols, number and diversity throughout country and good laboratory practice. Considering all difficulties, lack of laboratory guidelines and large number of Croatian laboratories performing general thyroid function tests, we hypothesize that there is heterogeneity in practice among them. The aim of this study was to estimate current laboratory preanalytical, analytical and postanalytical practices and policies on all levels of health care settings in order to make next steps toward quality improvement and tests harmonization.

## Materials and methods

### Methods

The WG for Laboratory Endocrinology of the CSMBLM designed a questionnaire regarding laboratory tests for the diagnosis of thyroid disease. The survey was sent in March 2020 through the web platform SurveyMonkey (SurveyMonkey Inc., Palo Alto, USA) to all medical biochemistry laboratories participating in external quality assurance (EQA) scheme for thyroid hormones (N = 111) organized by CROQALM. The participants were asked to submit a completed survey together with their EQA results. The survey was comprised of 27 questions/statements regarding preanalytical, analytical and postanalytical practices and protocols. Questions/statements were designed with one, multiple or descriptive answers.

### Statistical analysis

Answers to the survey are presented as absolute numbers and proportions. Fisher exact test was used to investigate differences in responses between primary health care and all other laboratories (grouped private health institutions, specialty hospitals, general hospitals, university hospitals and university hospital centres). Results were provided through statistical software MedCalc version 19.2.1 (MedCalc Software Ltd, Ostend, Belgium). Level of statistical significance was set at P < 0.05.

## Results

Out of 111 laboratories, 53 participants returned the questionnaire. Response rate varied depending on question, yielding a total survey response rate of 46-48%. Participating laboratories are from primary health care institution (21/53), specialty and general hospitals (17/53), university hospitals and university hospital centres (11/53) and private health care institution (4/53).

Study results, presented in Table 1, show diversity in performed thyroid tests, used analytical methods, EQA participations and providers. Since almost half of participants are from primary health care system, we compared answers of primary health care laboratories *vs* grouped all other types (private laboratories and all hospital types). Table 2 shows distributions of answers to questions/statements together with P values through all three phases of laboratory testing. The difference was observed for the location of blood sampling; sampling was done solely within the institution in 17/21 primary health care laboratories *vs* 17/32 grouped all others. Another difference was found in reporting test names; 3/20 primary care laboratories are reporting abbreviation together with full name *vs* 15/32 grouped all others. Most common type of reporting is abbreviations only, 17/20 primary care laboratories *vs* 15/32 all others. Only two (2/32) are reporting only full names. There were no statistically significant differences in other practices between these two groups.

**Table 1:** Frequency of performed analytes, used methods, external quality assessment participation and providers

	**N (proportion)**	
**Analyte**	**Performed**	**EQA participation**
Thyroid stimulating hormone	53 (1.0)	53 (1.0)
Free thyroxine	37 (0.70)	37 (0.70)
Total thyroxine	34 (0.64)	34 (0.64)
Free triiodothyronine	29 (0.55)	28 (0.53)
Total triiodothyronine	29 (0.55)	30 (0.57)
Thyroid peroxidase antibody	26 (0.49)	17 (0.32)
Thyroglobulin antibody	23 (0.43)	16 (0.30)
Thyroglobulin	5 (0.09)	5 (0.09)
Calcitonin	2 (0.04)	1 (0.02)
TSH receptor antibody	2 (0.04)	0 (0)
TSH-stimulating immunoglobulin	1 (0.02)	1 (0.02)
**Manufacturer (method)**	**N (proportion)**	
Abbott Architect/Alinity (CMIA)^1^	19 (0.36)	/
Beckman Coulter Advia/UniCell Dxl/Access2 (CLIA)^2^	11 (0.21)	/
Roche Elecsys/Cobas (ECLIA)^3^	10 (0.19)	/
Tosoh (FEIA)^4^	7 (0.13)	/
Siemens Centaur/Atellica (CLIA)^5^	3 (0.06)	/
Siemens Immulite (CLIA)^5^	3 (0.06)	/
Human (ELISA)^6^	1 (0.02)	/
Biomerieux Vidas (ELFA)^7^	1 (0.02)	/
Maglumi Snibe (CLIA)^8^	1 (0.02)	/
**EQA provider**	**N (proportion)**	
Croatian Centre for Quality Assessment in Laboratory Medicine, Croatia	53 (1.0)	/
Randox International Quality Assessment Scheme, United Kingdom	12 (0.23)	/
Labquality, Finland	4 (0.08)	/
Reference Institute for Bioanalytics, Germany	3 (0.06)	/
The European Society for External Quality Assessment, Germany	2 (0.04)	/
BioRad External Quality Assessment Services, United States	1 (0.02)	/
Institute for Quality Assurance Lübeck, Germany	1 (0.02)	/
United Kingdom National External Quality Assessment Service, United Kingdom	0 (0)	/
EQA – External Quality Assessment. TSH – Thyroid stimulating hormone. CMIA – chemiluminescent microparticle immunoassay. CLIA – chemiluminescent immunoassay. ECLIA – electrochemiluminescent immunoassay. FEIA – fluorescence enzyme immunoassay. ELISA – enzyme-linked immunosorbent assay. ELFA – enzyme-linked fluorescence assay. ^1^Abbott Diagnostics, Santa Clara, USA. ^2^Beckman Coulter, Brea, USA. ^3^Roche Diagnostics GmbH, Mannheim, Germany. ^4^Tosoh Corporation, Tosoh, Japan. ^5^Siemens Healthcare GmbH, Erlangen, Germany. ^6^HUMAN Gesellschaft für Biochemica und Diagnostica mbH, Wiesbaden, Germany. ^7^bioMérieux SA, Lyon, France. ^8^SNIBE - Shenzhen New Industries Biomedical Engineering, Shenzhen, China.		

**Table 2:** Frequency of answers to questions/statements and difference between primary care laboratories and all others

**Question/Statement**	**N (proportion)**	**P** (Primary care *vs* all others*)
**1. Blood sampling is performed:**		
Within institution	19 (0.36)	0.046
Both, within and other institution/location	34 (0.64)	
**2. The laboratory has defined patient preparation procedure for blood sampling:**		
Yes	29 (0.55)	1.000
No	24 (0.45)	
**3. Sample type used is:**		
Only serum	49 (0.92)	0.143
Serum and plasma	4 (0.08)	
**4. The laboratory records the use of suppression or replacement therapy:**		
Yes	17 (0.0.32)	0.137
No	36 (0.68)	
**5. The laboratory takes into account the TSH circadian rhythm:**		
Yes	17 (0.32)	0.374
No	36 (0.68)	
**6. The laboratory defines the thyroid function tests as:**		
Routine procedures only	47 (0.89)	0.384
STAT and routine procedures	6 (0.11)	
**7. The laboratory performs all tests ordered by the PCP (if signed contract with CHIF):**		
Yes	43 (0.90)	0.059
No	5 (0.10)	
**8. The lowest TSH reporting limit is defined with:**		
Limit of detection	31 (0.58)	0.399
Limit of quantitation	22 (0.42)	
**9. The laboratory verifies the limit of quantitation declared by the manufacturer for TSH:**		
Yes	6 (0.12)	0.664
No	46 (0.88)	
**10. The laboratory has defined a protocol for the detection of heterophilic antibodies interference:**		
Yes	9 (0.18)	0.137
No	42 (0.82)	
**11. The test name is reported as:**		
Full name and abbreviation	18 (0.35)	
Full name only	2 (0.04)	0.014
Abbreviation only	32 (0.62)	
**12. Test names are reported according to CCMB:**		
Yes	42 (0.86)	0.238
No	7 (0.14)	
**13. The assay method is recorded on the laboratory report:**		
Yes	48 (0.91)	0.074
No	5 (0.09)	
**14. The laboratory uses International System of units (SI) for reporting:**		
Yes	52 (1.00)	NA
No	0 (0.0)	
**15. The reference interval for adult population is:**		
Manufacturer-declared	48 (0.91)	1.00
Other literature or in-house derived	5 (0.09)	
**16. The laboratory provides age-specific reference intervals:**		
Yes	26 (0.49)	0.782
No	27 (0.51)	
**17. The laboratory reports gestation-specific reference intervals or cut-off values:**		
Yes	4 (0.08)	1.000
No	49 (0.92)	
**18. The laboratory verifies adopted reference intervals:**		
Yes	18 (0.36)	0.369
No	32 (0.64)	
**19. The TSH reference interval for adult population is reported as:**		
5^th^ and 95^th^ percentiles (90% reference interval)	6 (0.14)	
2.5^th^ and 97.5^th^ percentiles (95% reference interval)	24 (0.66)	0.521
0.5^th^ and 99.5^th^ percentiles (99% reference interval)	13 (0.30)	
**20. The laboratory defines minimal retesting interval:**		
Yes	4 (0.08)	0.143
No	49 (0.92)	
**21. The laboratory informs physician or patient about critical risk results defined by CCMB:**		
Yes	50 (0.94)	0.269
No	3 (0.06)	
*Grouped private health care institutions, specialty hospitals, general hospitals, university hospitals and university hospital centres. CCMB – Croatian Chamber of Medical Biochemist. CHIF – Croatian Health Insurance Fund. NA – not available. PCP – primary care physician. TSH – thyroid stimulating hormone.		

From nine laboratories that have defined protocols for the detection of heterophilic antibodies interference, five defined their current practice with simple protocol: two as pre-treatment with polyethylene glycol (PEG), two as serial dilutions and one with use of heterophilic blocking tube (HBT). Other four laboratories stated their protocol as different combinations: (i) serial dilutions and treatment with HBT, (ii) serial dilutions and measurement with other method, (iii) repeating measurement with the same and other method and (iv) pre-treatment with PEG, serial dilutions, treatment with HBT and measurement with other method. Only three laboratories provided minimal retesting interval (MRI): 30 days for TSH (3/53), T3 (3/53), T4 (3/53), fT3 (1/53) and fT4 (2/53).

## Discussion

Our study aimed to identify common practice regarding preanalytical, analytical and postanalytical procedures in thyroid testing among laboratories in Croatia. Great heterogeneity was identified for some important issues, mainly patient preparation, sensitivity of the TSH assay, reference intervals and interference management.

According to results in Table 1, not all participants use EQA schemes. Croatian Centre for Quality Assessment in Laboratory Medicine, as the only Croatian EQA provider, offers some of thyroid analytes (TSH, fT3, fT4, T3, T4 and Tg) in Modul 8 scheme and Croatian Chamber of Medical Biochemists obligates Croatian laboratories to participate in it. If analytes are not offered by national EQA provider, laboratories must use other international providers (*11*). There is 100% participation for TSH, fT4, T4, Tg and TSI, while there is disproportion of answers (performed *vs* EQA enrolment) for fT3 and T3. As CROQALM offers fT3 and T3, we believe this to be unintentional error during answering of questionnaire. The main reason for concern is specialized thyroid tests (anti-TPO, anti-Tg, hCT and TRAb) where not all laboratories performing these tests are using EQA schemes. Table 1 also shows great heterogeneity with nine different immunoassay methods used. Many studies clearly demonstrate marked variations in measured thyroid hormones concentration between analytical platforms, as confirmed by Barth *et al.* and Strich *et al.* (*12*, *13*). This heterogeneity of used methods and their poor standardization, lead to incomparable results between laboratories.

Results of our survey showed almost half of laboratories do not provide any special instructions for thyroid testing (*e.g.,* use of suppression or replacement therapy before blood sampling, circadian rhythm). This is even more alarming knowing that 2/3 of participants declared blood sampling outside their institution/location. More hospital and private than primary health care laboratories are collecting blood samples outside their institution. This is not a surprise as hospital and private laboratories often provide bigger test menu of specialized tests and are referral institutions (*2*). Lack of adequate instructions for patient preparation, especially in the field of endocrinology was previously also established by the WG for Patient Preparation of the CSMBLM in 2015 (*14*). Measured concentrations can be influenced by different preanalytical issues as fasting, circadian rhythm, sleep deprivation, acute and chronic stress (*15*–*17*). As management of thyroid disease includes substitution therapy (most common levothyroxine), patients should have instructions regarding time of intake of medication, provided by the laboratory or primary care physician (PCP). Management of non-thyroid conditions (with medications such as amiodarone), intake of any kind of iodide, either as supplements or part of diagnostic test (*e.g.,* radiopaque dyes) influences thyroid hormone concentration (*18*, *19*). All listed factors should be considered, and laboratories should provide clear instruction to the patients on avoidance of it before the laboratory testing.

Based on our results, analytical interferences seem to be under recognized since only small proportion of laboratories have implemented protocols for dealing with heterophilic antibody interferences. There are many reports of interference of heterophilic antibodies (human anti-mouse antibodies (HAMA) or human anti-animal antibodies (HAAA)) that caused serious diagnostic errors by producing falsely elevated or decreased hormone concentration (*20*, *21*). It is therefore necessary to suspect possible interference when clinically unexpected result is observed and apply one of several protocols for managing interferences: pre-treatment with PEG, serial dilutions, treatment with HBT or measurement using different methods.

Several postanalytical reporting issues emerged, such as test names, the lowest limit of measured concentration, and reference interval. Reporting test names (full name, abbreviation, or both) showed the difference between healthcare settings, but every combination is accepted according to national recommendations by Krleža *et al.* (*22*).

For some analytes, accurate and reliable measurement of low concentration is of clinical importance. The majority of participants use the limit of detection (LOD) as the lowest value of reported TSH, while less than half use the limit of quantitation (LOQ). The reason for concern is that only six participants are verifying manufacturer-claimed LOQ. Most currently available TSH immunoassays have a third-generation claim with a functional sensitivity of ≤ 0.02 mIU/L (*23*). Third-generation performance is required for detecting subclinical hyperthyroidism or adjusting suppressive doses more carefully in patients receiving exogenous thyroid hormone (*24*-*26*). Therefore, there is a clinical utility in reliable measuring of TSH concentrations between 0.01 and 0.1 mIU/L. The limit of quantitation should be used as the lowest reportable limit for measured concentration above LOD and below LOQ. The limit of detection should be reported only when the measured concentrations are below the limit of blank (LOB) (*27*).

Although most laboratories (91%) adopt the reference interval for adult population from manufacturer’s instruction for use, reporting of TSH reference intervals significantly differs between them with reference intervals defined as 90, 95 or 99%. There is no single recommendation on range of reference intervals, although most common in use is 95% (*28*). Only one third of laboratories are verifying adopted reference intervals before implementing them in routine practice, which is consistent with report from WG for Postanalytics of the CSMBLM (*29*). This is surprising since verification is obligatory according to Croatian Chamber of Medical Biochemists and international standard ISO 15189 (*30*). Almost 50% of laboratories in the study do not provide age-specific reference intervals and almost none report gestation-specific reference intervals. Children are undergoing hormonal maturation until the end of puberty, which requires use of age-specific reference intervals. Additionally, during pregnancy reference interval should be listed according gestation weeks. Unfortunately, most manufacturers do not provide reference intervals for children and pregnant women and many studies are done in order to fill this gap (*31*–*33*). All these issues complicate the interpretation and comparability of laboratory results for TSH and there is an urgent need for standardization of thyroid hormone assays to reduce inter-laboratory variation.

Our study has some limitations. Firstly, response rate was low and only half of laboratories that are performing thyroid testing participated in our study. In order to cover all phases of laboratory work, survey was rather long which might have discouraged some participants. Some might not be inclined to share their work practices, especially when they are not in accordance with good laboratory practise. In addition, since this was a self-fulfilling survey, we cannot exclude the possibility that participants gave desirable answers, rather than reporting exact practice in their laboratories.

This study showed current preanalytical, analytical and postanalytical laboratory practices and helped to identify some critical spots in thyroid testing in Croatia. It is certainly going to serve as a foundation in creating National guidelines, which will help harmonizing laboratory procedures in thyroid testing.
